# T Cell engineering for cancer immunotherapy by manipulating mechanosensitive force-bearing receptors

**DOI:** 10.3389/fbioe.2023.1220074

**Published:** 2023-07-25

**Authors:** Lingzhu Zhao, Guoqing Zhao, Jinteng Feng, Zheng Zhang, Jiayu Zhang, Hui Guo, Min Lin

**Affiliations:** ^1^ The Key Laboratory of Biomedical Information Engineering of Ministry of Education, School of Life Science and Technology, Xi’an Jiaotong University, Xi’an, China; ^2^ Bioinspired Engineering and Biomechanics Center (BEBC), Xi’an Jiaotong University, Xi’an, China; ^3^ Department of Thoracic Surgery, First Affiliated Hospital of Xi’an Jiaotong University, Xi’an, China; ^4^ Department of Medical Oncology, First Affiliated Hospital of Xi’an Jiaotong University, Xi’an, China

**Keywords:** mechanobiology of T cell, T cell activation, immunoengineering, cancer immunotherapy, mechanosensitive receptors

## Abstract

T cell immune responses are critical for in both physiological and pathological processes. While biochemical cues are important, mechanical cues arising from the microenvironment have also been found to act a significant role in regulating various T cell immune responses, including activation, cytokine production, metabolism, proliferation, and migration. The immune synapse contains force-sensitive receptors that convert these mechanical cues into biochemical signals. This phenomenon is accepted in the emerging research field of immunomechanobiology. In this review, we provide insights into immunomechanobiology, with a specific focus on how mechanosensitive receptors are bound and triggered, and ultimately resulting T cell immune responses.

## 1 Introduction

**FIGURE 1:**
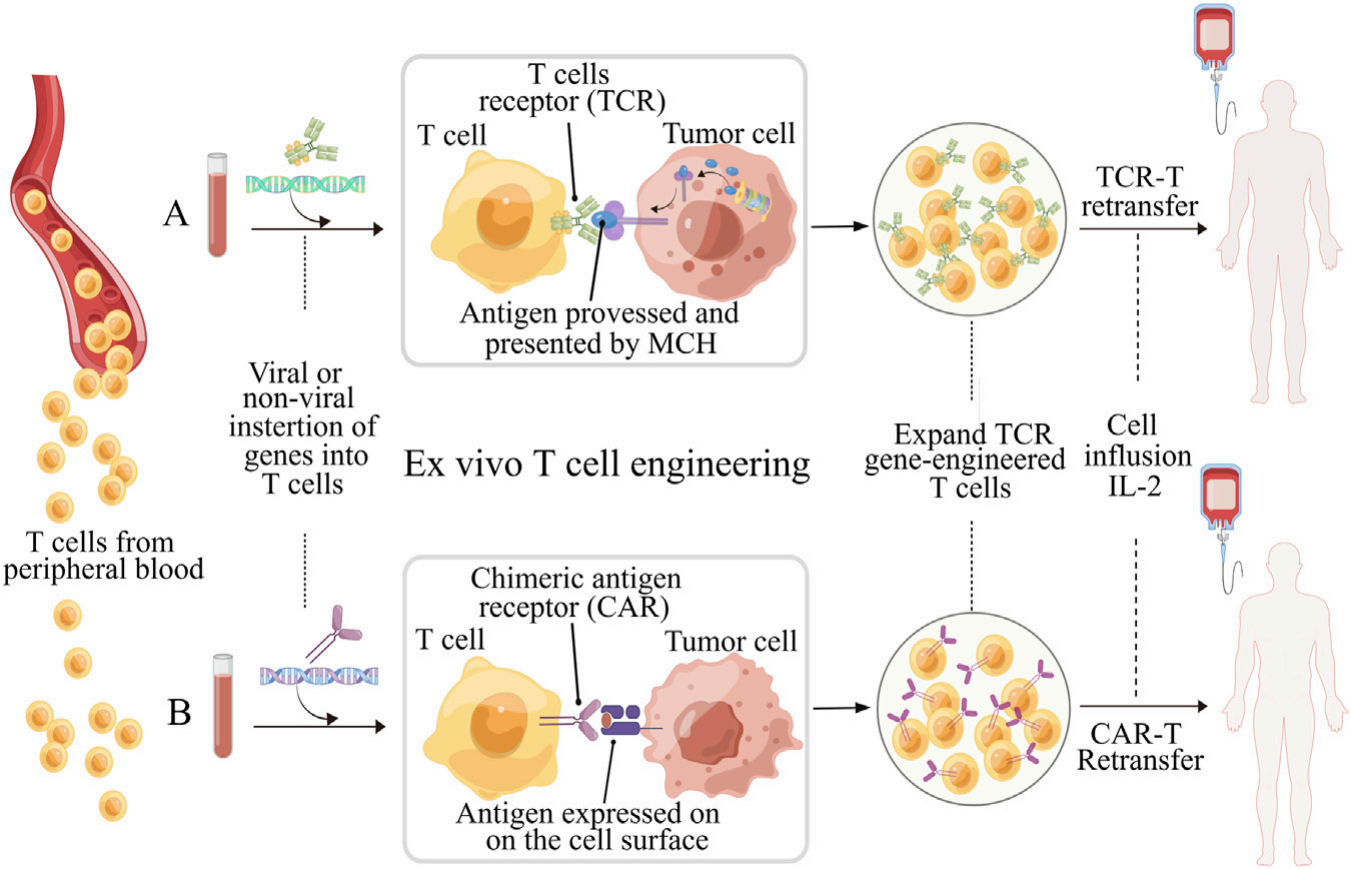
Schematic illustration of *ex vivo* T cell engineering for adoptive cell therapy (ACT). TCR-T and CAR-T cells, which are genetically modified peripheral blood cells designed to recognize tumor antigens and enhance the effectiveness of ACT. Prior to reinfusion, both types of T cells require activation (using anti-CD3/CD28 beads) and expansion. TCRs and CARs have different molecular structures and recognize different antigen peptides. **(A)** TCR consists of an α chain and a β chain, and recognize peptides the antigens processed and presented by MHC molecules. Transgenic TCRs have the potential to target a wide range of tumor antigens since both intracellular and surface proteins can be presented as peptides in the context of MHC. **(B)** CARs are artificial receptors composed of variable regions of the heavy and light chains of antibody associated with intracellular signaling chains, such as CD3-ζ, CD28 or 41BB. The antigens recognized by CARs do not need to be restricted by MHC molecules, but must be presented on the surface of tumor cell. Figure is obtained by Figdraw.

Over the past decade, there has been significant progress in the field of immunotherapy, which aims to modulate immune responses for disease therapy (Del Paggio, 2018; Milone et al., 2018). Adoptive T cell therapy (ACT) has appeared as a promising approach to fight against cancers and other diseases, and has even obtained outstanding curative efficacy in some patients with refractory or relapse-prone cancers (June 2007). However, the clinical responses of ACT for the bulk of cancer patients remain confined and challenging due to various factors. These include the difficulty in isolating and expansion functional tumor-specific T cells *ex vivo*, T cells exhaustion resulting from high tumor antigen burden, and diverse mechanisms of immune suppression induced by tumors (Hegde and Daniel, 2020; Wong et al., 2022). To achieve immunotherapies based on T cell with improved efficacy and indispensable safety, massive efforts have been committed to engineering T cell with biochemical signals, such as chimeric antigen receptor (CAR), transgenic T cell receptor (TCR) for desired specificity (Figure 1) (Met et al., 2019). Table 1 lists the six CAR-T cell therapy currently approved by the FDA. The use of CAR-T cell receptor in cancer treatment has been an active research area with both experimental and computational studies in recent years. This is mainly due to the increasing use of various types of CAR-T cell receptors, such as single-molecule receptors and biocompatible.

**TABLE 1:** CAR-T cell therapies approved by the FDA.

Drug	Target	Indications	Complete response (CR)	Company	Time to market
Kymriah	CD19	ALL	>90%	Novartis	2017.2
Yescarta		DLBCL	51%	Gliead	2017.10
		FL			
Tecartus		MCL	67%	Gliead	2020.7
Breyanzi		DLBCL	54%	BMS	2021.2
Abecma	BCMA	MM	28%	BMS/Blue bird	2021.3
Carvykti		MM	78%	Legend/Janssen	2022.2

FDA, food and drug administration. CD19, Cluster of differentiation 19; BCMA, B cell maturation antigen. ALL, acute lymphoblastic leukemia; DLBCL, diffuse large B cell lymphoma; FL, follicular lymphoma; MCL, mantle cell lymphoma; MM, multiple myeloma.

**FIGURE 2:**
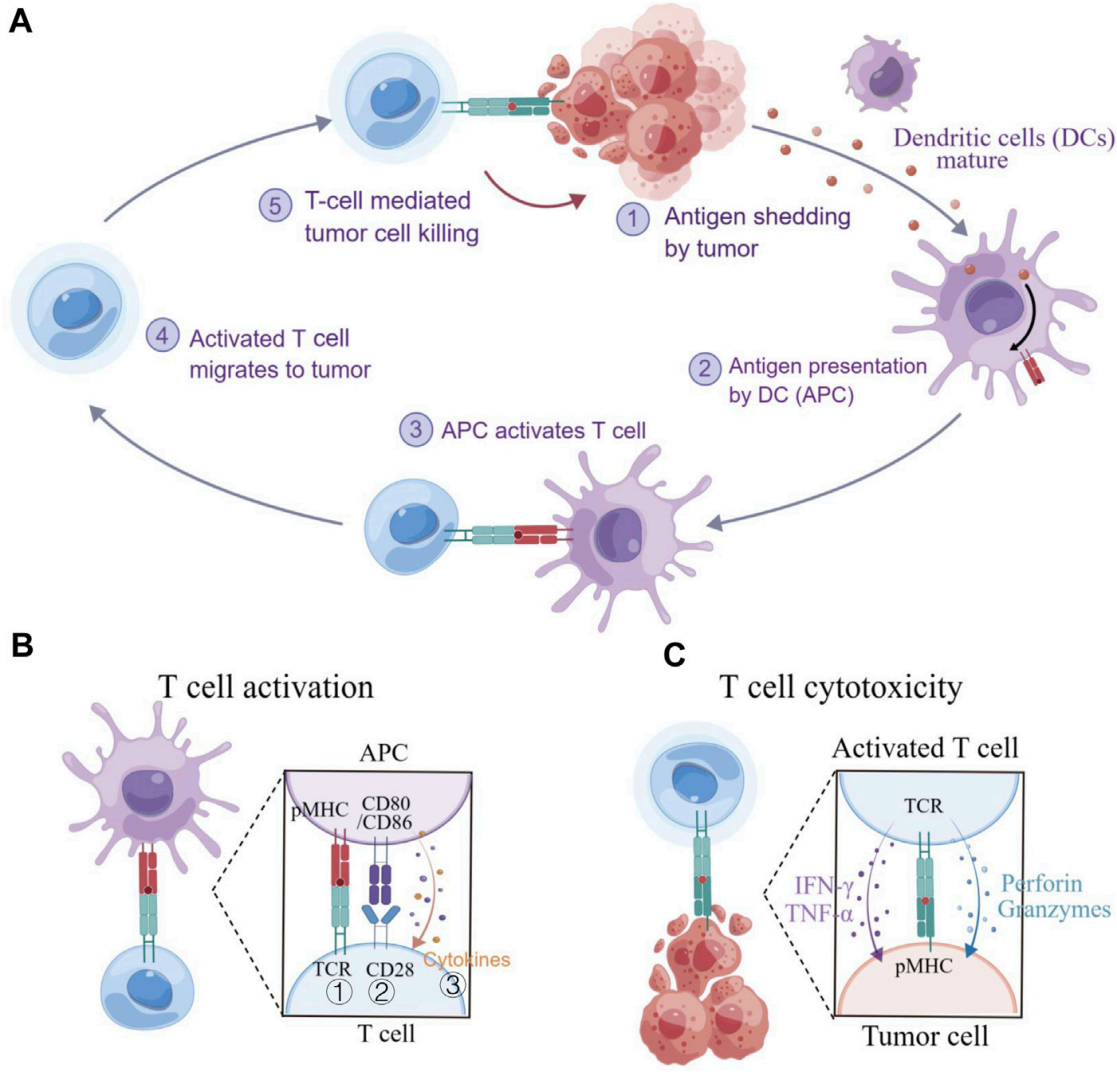
T cell immunity to cancer. **(A)** The cancer-immunity cycle involves several steps, including exposure or release of tumor antigens, tumor antigen processing and presentation by APCs, priming and activation of T cells, migration and infiltration of T cells to tumor tissues, and the recognition and killing of tumor cells. **(B)** T cell activation requires three signals: ① an initial stimulatory signal between the TCR and pMHC; ② co-stimulatory signal between CD28 and CD80/CD86, promoting T cell proliferation; and ③ the present of cytokines that support T cell differentiation. **(C)** Cytotoxic T lymphocyte (CLT)-mediated killing of tumor cells. CTLs recognize and eliminate cancer cells that present specific antigens. Figure is obtained by Figdraw.

T cells could be engineered to recognize and respond to disease states, making them as a promising “living drug” for patients. However, cancer-immunity interactions (Figure 2A) involve not only biochemical but also substantial biomechanical signals, which have often been overlooked in the past (Chen and Zhu, 2013). The diverse biomechanical microenvironment and varying biomechanical properties of tissues even cells under disease conditions (Cukierman and Daniel, 2010) could have been a significant effect on T cell functions, including development, activation, differentiation, migration, effector functions, and metabolism, etc., which are regulated by mechanosensitive force-bearing receptor-ligand interactions (Rowley et al., 2019; Guimarães et al., 2020). Therefore, considering the biomechanical factors may provide new perceptions into the design of T cell-based immunotherapy and improve their efficacy in treating diseases.

Although the impact of biomechanical factors on T cell immunity has been intensively studied in the context of biochemical signaling, their full integration into the design of immunotherapies has yet to be achieved due to limited understanding of their potential regulatory roles in diseases (Huse, 2017). Nonetheless, the nascent field of mechano-immunology has enabled the progress of novel engineering approaches that modulate ctions through an approach known as “mechanical immune engineering” (Lei et al., 2020). By combining biomechanical signals with new therapeutic approached, mechanical engineering of T cell immunity can design T cell immune responses and improve therapeutic efficacies (Li et al., 2020). In this review, we first discuss the role of mechanosensitive force-bearing receptors within the development of mechanical immune engineering approaches. We then summarize recent advantages in this field and emphasize their potential applications in cancer immunotherapy. Lastly, we provide insight into the future directions of mechanical immune engineering and its potential clinical therapeutic applications.

## 2 Mechanobiology of T cell activation

### 2.1 Mechanotransduction of TCR is vital to T cell activation

**FIGURE 3:**
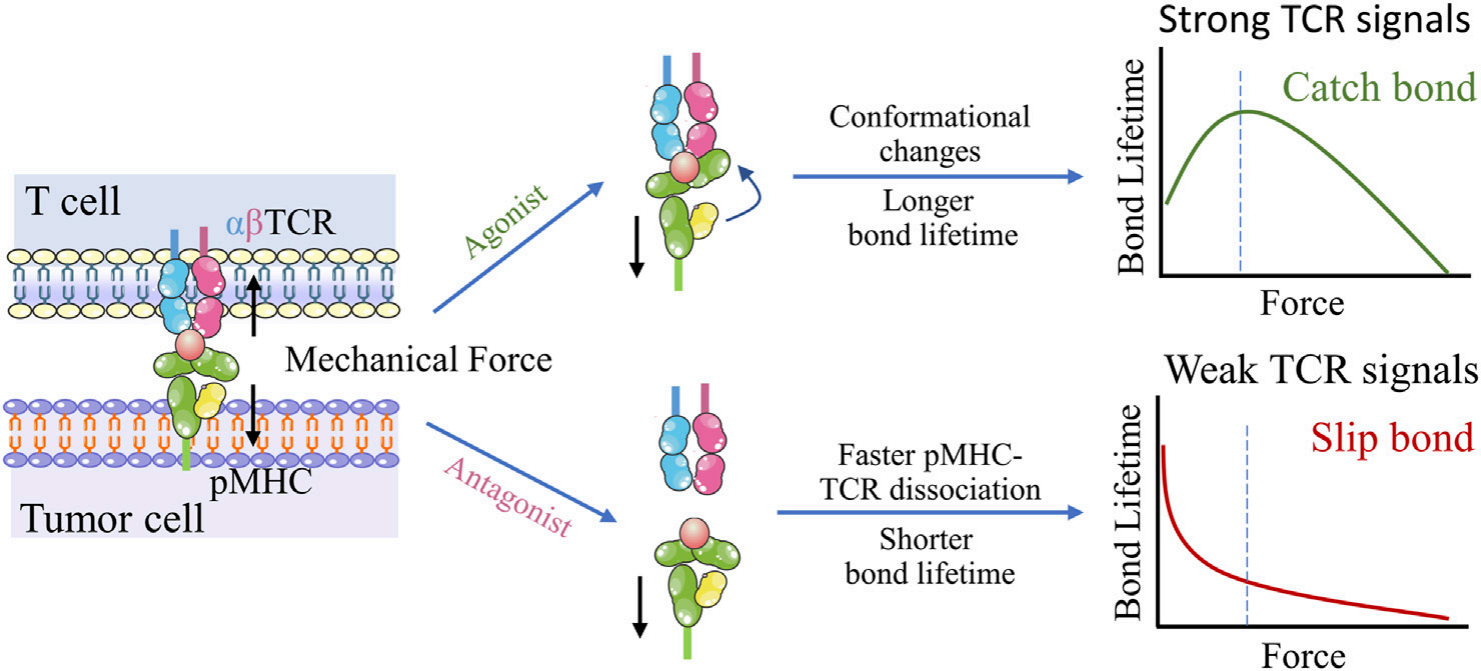
Dynamic structural mechanism of mechano-chemical coupling for TCR antigen recognizing. Upon application of mechanical force, the conformational change occurs in the agonist pMHC, which allosterically activates TCR-pMHC catch bonds, resulting in TCR antigen recognition and triggering of T cell signaling. In contrast, TCR interactions with low-affinity peptides exhibit slip-bond behavior, suggesting easy rupturing under low tensile forces.

In principle, adoptive cell therapy (ACT) requires T cell activation, which is governed by three signals (Figure 2B) (Huppa and Mark, 2003): 1) an essential stimulation signal by specific antigen combined with the TCR/CD3 complex for initial activation; 2) the co-stimulatory signal by CD28 for triggering T cell proliferation; and 3) autocrine or paracrine cytokines for T cell differentiation. Optimized conditions are required for each step to achieve the best therapeutic outcome (Waldman et al., 2020). While T cell activation has been known as a purely biochemical process, recent evidence suggests that mechanical forces can significantly enhance T cell activation by causing allosteric effects in the TCR/CD3 complex, which acts as a mechanosensor for T cells (Liu et al., 2014). The TCR can bear 12–19 pN of force in the time of encountering with antigen peptide-bound major histocompatibility complex (pMHC) (Liu et al., 2016; Ma et al., 2019), contributing to the specific sensitivity response of TCR triggering, and the discrimination to agonist quality and affinity (Wu et al., 2019). The simplified pattern of force-dependent TCR signaling is shown in Figure 3.

The TCR plays a critical role as a mechanosensitive receptor of T cell within the early stage of triggering activation, relying on the intracellular actin cytoskeleton for proper functions (Hu et al., 2016; Butte et al., 2017). Through the TCR, T cells are able to sense external mechanical properties such as substrate stiffness and spatial arrangement of ligands (Zhu et al., 2019). Interestingly, TCR exhibits a biphasic response to substrate stiffness, which is distinct from other well-known mechanosensitive receptors like integrin (Wahl et al., 2019; Yuan et al., 2021). Upon mechanical activation of the TCR, cytoskeletal forces are generated via polymerization and rearrangement of actin, and retrograde actin flow mediated by myosin-II (Hui et al., 2015; Hu and Butte, 2016), allowing for the connection between T cells and their environment on account of cytoskeletal forces transmitting to antigen-presenting cells (APCs) or target cells via the transmembrane domain of TCR (Judokusumo et al., 2012; Colin-York et al., 2019). While inhibition of the cytoskeletal forces leads to restraint of T cell activation occurrences, such as sharp reduction of interleukin-2 (IL-2) production. Additionally, recent studies have shown that the exertion of mechanical forces against cancer cells by cytotoxic T lymphocytes (CTLs) through immunological synapse, promote killing to cancer cells via enhancing pore formation induced by the perforin protein into the membrane of cancer cells (Basu et al., 2016). Furthermore, the membrane protrusions of CTLs at F-actin-rich regions have been demonstrated to be essential for synaptic mechanical force output and efficient cytotoxicity against target cells (Huse, 2019; Jin et al., 2019). T cell mechanical signals are force-dependent triggered by TCR/CD3 complexes upon binding to pMHCs or anti-CD3 antibodies (Hu et al., 2016). Recently, Tang’s group developed a force-triggered system that is responsive to cellular force of T cell for drugs delivery, releasing the drug upon TCR triggered (Lei and Tang, 2020). These anticancer drugs delivery design based on T cell mechanical signal has been shown to significantly potentiate the destruction to cancer cells both *in vitro* and vivo, demonstrating the potential for T cell mechanical force that can coordinate to controlled drugs delivery by precisely releasing of desired molecules for enhanced therapeutic efficacy. These evidences indicate that mechanical forces are crucial for not only T cell activation, but also for T cell mediated cytotoxicity.

### 2.2 Integrins mediated T cells response via mechanotransduction

Integrins play a critical role in regulating cell proliferation, adhesion, and migration by transmitting mechanical and chemical signals between extracellular environment and the cell interior (Saraswathibhatla et al., 2023). Upon binding to the ligands in extracellular matrix (ECM), force generation promotes integrin activation and integrin clusters forming, which can allow translation of mechanical force to the cytoskeleton via a series of mechanical sensitive adaptor proteins (Kechagia et al., 2019). These effects induce the rearrangements of actin cytoskeleton and the formation of focal adhesions, such as actin bundling and stress fibres. These rearrangements, in turn, activates intracellular signalling pathways (Cosgrove et al., 2016). Integrins also have vital function in cell migration, with integrin-mediated molecular clutch mechanisms proposed to explain the relationship between cell migration speeds and ECM rigidity in haptotaxis and durotaxis (Bangasser et al., 2017; Isomursu et al., 2022). Recent studies have identified integrin signaling as a key driver of multiple cell functions, including stem cell differentiation (Zhang C et al., 2021; Zhang et al., 2022), tumor initiation, epithelial plasticity, metastatic reactivation, and immune-targeted therapies, etc. (Pang et al., 2023). Integrins are also as force sensors that actively modulate T cells effector functions (Comrie et al., 2015b). For instance, in response to high substrate rigidity, integrin lymphocyte function-associated antigen-1 (LFA-1) undergoes a conformational change which can improve the affinity with immobilized intercellular adhesion molecule-1 (ICAM-1), facilitating T cell adhesion and priming (Li et al., 2009). This process occurs during dendritic cells (DCs) maturation (Comrie et al., 2015a). Recent studies show that LFA-1/ICAM-1 interactions can facilitate close and physically active contact between the CLTs and target cells, resulting in enhanced degradation and cytotoxic secretion, thus contributing to the potency and specificity of killing target cells (Wang et al., 2022).

**FIGURE 4:**
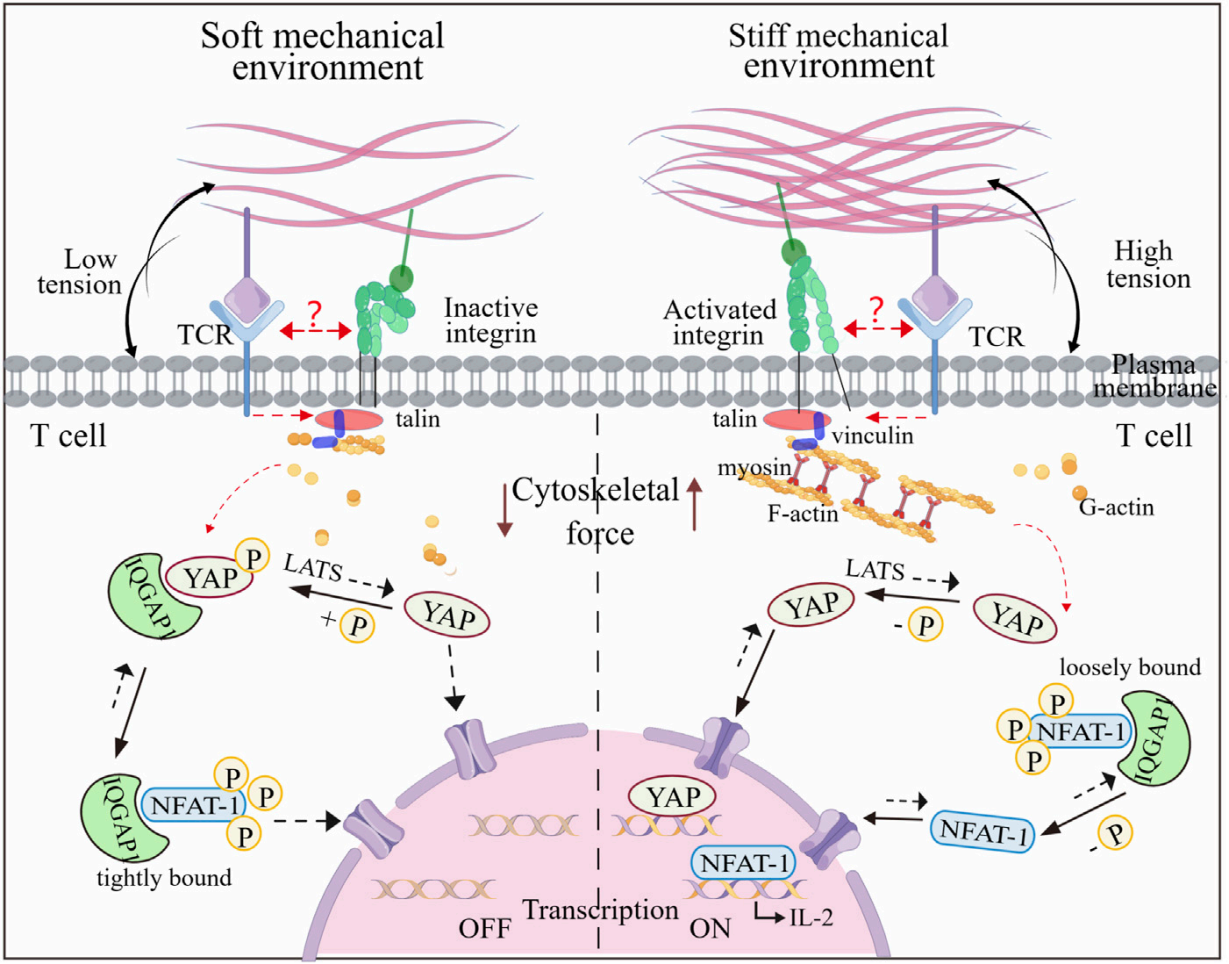
Mechanoregulation of T cell activity mediated by nuclear shuttling of YAP and NFAT-1. The T cell activity to stiffness depends on gene regulators such as YAP and NFAT-1, which can shuttle between the cytoplasm and the nucleus under mechanical cues. In a soft microenvironment, YAP is phosphorylated by LATS and immobilized in the cytoplasm, interacting with IQGAP1. This interaction enables IQFAP1 to bind tightly with NFAT1. The nuclear localization of NFAT1 is thus impaired, which attenuates cellular activation and metabolism. In contrast, YAP is dephosphorylated and transfers to the nucleus when T cells experience the stiff microenvironment, which promotes F-actin polymerization and integrin-mediated mechanosensing. Additionally, NFAT1 can dissociate from the NFAT1-IQFAP1 complex, allowing it to be dephosphorylated and transported into the nucleus, thereby inducing expression of genes associated with T cell activation. It is unknow whether TCR and integrin can interfere with the YAP and NFAT-1 signaling events. Figure is obtained by Figdraw.

Recent research has shown that RGD immobilized on stiff substrates enhances integrin-mediated signaling from the ECM, facilitating F-actin assembly and actomyosin contractility, leading to cytoskeletal tension, and nuclear localization of Yea-associated protein (YAP), as a mechanotransducer based on mechanical signals of extracellular matrix (Vining and David, 2017). Similar stiffness-mediated cellular localization of YAP has exposed in T cells (Elosegui-Artola et al., 2017). Phosphorylated YAP plays a role in modulating T cell activation based on matrix stiffness by regulating the binding affinity between IQGAP1 (Ras GTPase-activating-like protein) and NFAT-1 (nuclear factor of activated T cells-1), affecting the T cell activation and metabolism by promoting IL-2 secretion (Meng et al., 2020). YAP stabilizes interactions of IQGAP1 and NFAT-1 in the cytoplasm by suppressing NFAT-1 from nuclear localization in response to low ECM rigidity via T cell adhesion to RGD that is immobilized on hydrogels, attenuating metabolic reprogramming and T cell activation. In contrast, on high ECM rigidity, most cytoplasmic YAP translocates into the nucleus, thus promoting NFAT-1 transportation into the nuclear and upregulating proliferation and metabolism. Therefore, YAP mediates the restriction of T cell responses, once the mechanical environment transitions from high rigidity (inflammation) to low rigidity (baseline) (Figure 4). This stiffness-dependent suppression also preserves healthy tissues from attacking by T cell-mediated autoimmunity. Overall, this “mechanical checkpoint” behavior-mediated the special mechanical properties of T cells may present a novel anchor for engineering optimal T cell effector responses.

### 2.3 Inhibitory receptor/ligand regulates T cells through force-dependent interactions

T cells upregulate the expression of co-inhibitory receptors, also called as immune checkpoints, on their membrane when exposed to the tumor microenvironment. These immunosuppressive receptors regulate T cell immunity negatively, including programmed cell death protein 1 (PD-1), cytotoxic T-lymphocyte-associated protein 4 (CTLA-4), and mucin domain-3 (TIM-3), etc., which can reduce T cell activity and inhibit their ability to proliferate and produce cytokines (Wherry and Kurachi, 2015). These inhibitory signals present significant challenges for ACT (Curdy et al., 2019). Recent findings suggest a possible association between immune checkpoints and mechanotransduction. Salaita et al. reported a DNA probe that can preserve the mechanical information generated by T cells and reveal the role of mechanical force in modulating the immune checkpoint. Additionally, their research revealed that T cells exert force on anti-PD-1 antibody, through the PD-1 receptor (Ma et al., 2019).

Immune checkpoint inhibitors (ICIs) can conquer the inhibitory signals to re-activate exhausted T cells (Rumpret et al., 2020). Monoclonal antibodies (mAbs) targeting PD-1/PD-L1 (Programmed cell death 1 ligand 1) have remarkably innovated cancer therapy through immune checkpoint blockade. Chen et al. developed an ultra-stable bio-membrane force probe and benchmarked the dissociation kinetics of three clinically approved PD-1 blockade mAbs (An et al., 2020), which correlated well with the objective response rates in cancer clinical treatment. This platform potentiates the screening, optimization, and clinical selection of therapeutic antibodies in the future. Moreover, a recent study revealed that cancer cells deplete CD80 and attenuate T cell activation by utilizing force-dependent trans-endocytosis. Forces induced by cancer cells are transmitted through the binding of CTLA-4 and CD80 (Park et al., 2020). The generation of inhibiting force in cancer cells could facilitate the activating capacity of APCs to T cells (Oh et al., 2020; Peng et al., 2020). This may inspire a new strategy to rescue anti-tumor immunity through inhibiting force-generation. However, further research is required to explore the mechanics of force-responded immune checkpoints.

Mechanotransduction refers to the conversion of mechanical signals into biochemical signals that activate cellular pathways and regulate cell function. T cells, in particular, heavily rely on rearrangement in their cytoskeletal structures in response to mechanical cues for functions such as activation, immunological synapse formation, immune reactivity, and migration. Understanding mechanosensing pathways and actin function in T cells could offer the potential to predict and guide clinical applications. As researchers sheds more light on nuclear actin (Irvine et al., 2021), there is growing interest in modulating T cell activation and differentiation as well as enhancing or restraining the immune response through considering actin dynamics.

## 3 Mechanical immunoengineering strategies for T cell immunity

### 3.1 Stiffness dependent T cells mechanoimmunology

The mechanical properties of tissues play a critical role in modulating immune function, and the diseased tissues tend to suffer from diverse ECM properties in contrast to the healthy tissues. For instance, *in vivo* mouse models, the stiffness of sentinel lymph nodes in inflamed tissue becomes up to 40 kPa, which is stiffer than normal lymph nodes (only about 4 kPa) (Meng et al., 2020). Such changes in mechanics of lymph node tissue have been demonstrated to affect T cell activation, with T cells exhibiting increased activation on stiffer ECM, which is mediated by YAP mechanosensing (Figure 4). To better understand the impact of mechanical properties on T cell activation and differentiation, researchers have been employed 2D hydrogels with varying stiffness (Yuan et al., 2021). These studies have shown that TCR and LFA-1have differential contributions to T cell spreading on different stiffness, with TCR regulating a biphasic response, and LFA-1 showing a monotonous response upon co-engagement. These results indicate that T cells distinguish from different substrate stiffnesses *in vivo* and adjust their behaviors correspondingly.

Efforts have been devoted to leverage the stiffness responsiveness of T cells to improve cancer immunotherapy. For instance, researchers have designed an artificial T cell stimulating matrix (aTM) hydrogel based on ECM materials with mutable stiffness and T cell stimulation signals. The aTM has been shown to potentiate mechanically sensitive TCR signaling, resulting in a rapid and robust expansion of T cells and effective immunotherapy (Hickey et al., 2019). This approach not only holds immediate immunotherapeutic applications, but also provides a roadmap for the future engineering ECM-simulate materials for therapeutic T cell activation. Overall, these findings underscore the significance of understanding the impact of tissue mechanics on immune function and suggest that engineering tissue-simulate materials can offer a promising strategy for therapeutic immune modulation.

**FIGURE 5:**
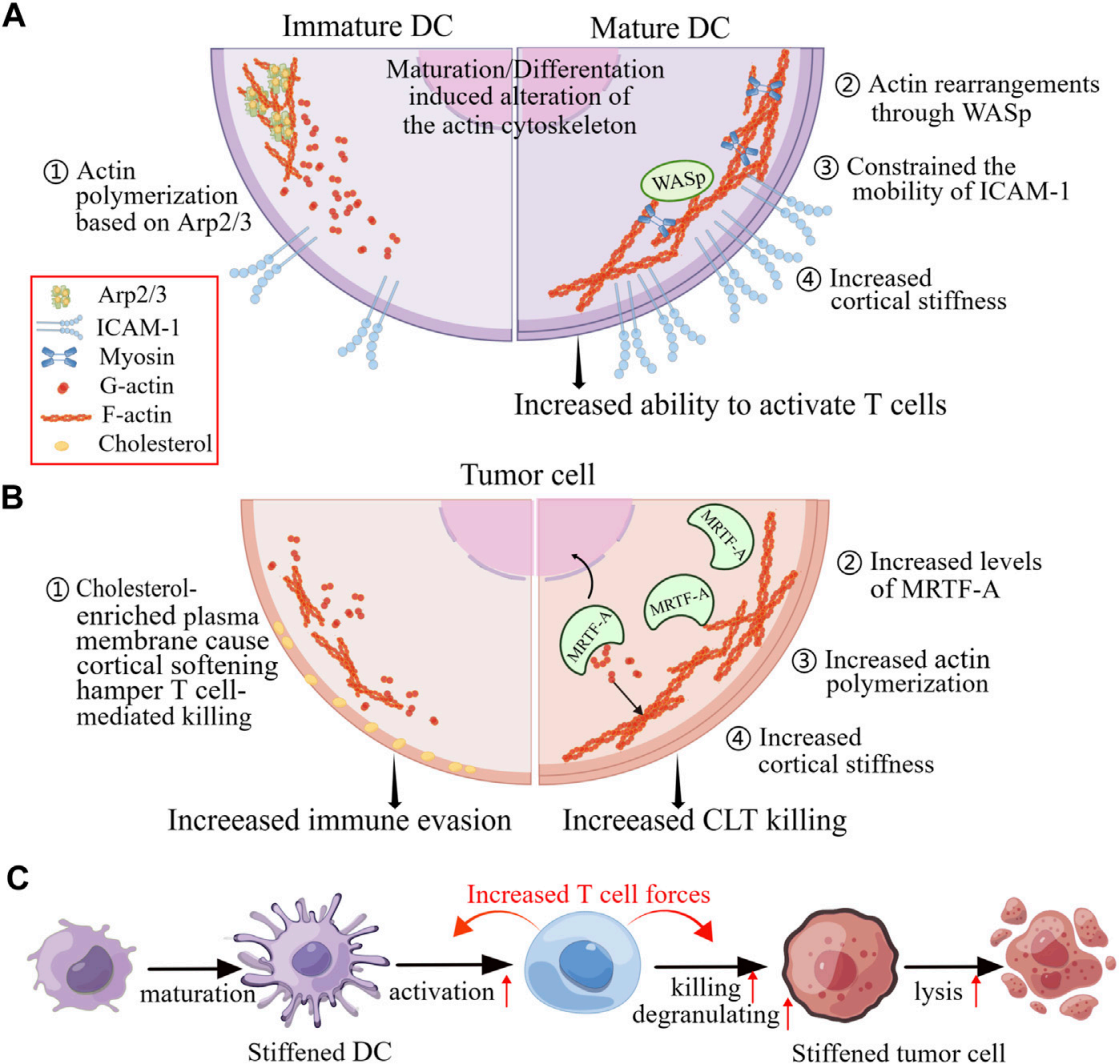
Changes in mechanical properties of cells in response to physiological and pathophysiological alterations affect cell-to-cell contact. **(A)** The maturation of DCs and changes in actin polymerization pathways can enhance the activation of T cells by increasing the confinement of membrane proteins and the rigidifying of the cortex of the DCs. **(B)** The killing of T cells to tumor cells by is impeded when the tumor cells are cortically soft due to an enrichment of cholesterol in their plasma membranes. Conversely, increased levels of MRTF and actin content lead to increased cortical rigidity, making tumor cells more susceptible to cytotoxic T cells. **(C)** When DCs and tumor cells are stiffened, they become better at activating T cells and being killed by T cells, respectively. These enhanced functionalities are due to the accumulation of F-actin at immunological synapse, which generates cellular forces exerted by T cells. Figure is obtained by Figdraw.

Additional studies have revealed the cellular rigidity acts a significant role in modulating T cell responses through cell-cell interactions. As the major professional APCs, DCs are particularly effective in priming T cell responses. Actin rearrangements in both DCs and T cells characterize the DC-T cell immunological synapse (Leithner et al., 2021). Actin rearrangements through Wiskott-Aldrich syndrome protein (WASp) and Hem1 containing WASp-family verprolin-homologous protein (WAVE) complexes promote the appearance of podosomes on the DC side, which are thought to increase rigidity and promote maintenance of cell contact, thereby enhancing T cell priming and activation (Figure 5A) (Malinova et al., 2015).

The malignant and progression of tumors have been found to be associated with increased cancer cells rigidity. Researches have shown that cancer cells cultured on stiff substrates are more vulnerable to cytotoxicity of CTLs, and T cells exhibit greater activity coculturing with the target cells which are grown on stiffer substrates, indicating that the biomechanics of target cells can affect T cell immunotherapies (Basu et al., 2016). Furthermore, increasing cellular rigidity of cancer cells through overexpression of the myocardin related transcription factor (MRTF) have been found to reduce their metastatic colonization and enhance responsiveness to CTL-mediated killing (Figure 5B) (Tello-Lafoz et al., 2021).

Similarly, Tang et al. demonstrated that cholesterol depletion-induced cancer-cell stiffening increased T cell cytotoxicity and improved the efficacy of adoptive T cell therapy against solid tumors in mice. This was due to increased T cell forces caused by the constantly amass of filamentous actin in immune synapse (Figure 5B) (Lei et al., 2021). However, softening cell prevents cytolytic T cell killing of tumor-repopulating cells (TRC) (Liu et al., 2021). The softness of TRCs prevents the adequate generation of contractile force for perforin pore formation, as the interaction of perforin with non-muscle myosin heavy-chain 9 in soft TRCs results in reduction of F-actin. Consequently, perforin released by the TRC softness is unable to create membrane pores, leading to attenuated cytotoxicity and increased immune evasion. This process of T cells sensing biomechanical vulnerability is called “mechanical immunosurveillance”, which reveals a potential role of biomechanical properties in T cell immunity and provide a primary direction for mechanical immune engineering of T cells.

### 3.2 Topography and ligand presentation dependent T cell mechanobiology

Optimizing T cell activation and expansion *in vitro* is an important therapeutic approach toward for enhancing immune response to specific antigens (Eggermont et al., 2014). As such, the development of engineered surfaces for optimizing the activation and expansion of T cells are crucial (Babensee et al., 2015). The geometry of artificial antigen-presenting cells (aAPCs) acts a key role in modulating the effective binding of ligands with T cell membrane by triggering more contact area and frequency. For example, it has been shown to promote higher proliferation of cytotoxic T cells *in vivo* and *ex vitro* by stimulated with ellipsoidal aAPCs, compared to their spherical counterparts, even at similar antigen density (Meyer et al., 2015).

Another promising approach for T cells expansion involves attaching T cell ligands to the artificial substrates with high surface area. For instance, bundled carbon nanotubes can provide a high density of surface stimulation, promoting generation of abundant cytotoxic T cells (Fadel et al., 2014). In this case, the proliferation of cytotoxic T cells was significantly improved by controlling the release of IL-2 from these nanotubes. Additionally, the nanoparticles with stimulating antibodies to CD3 and CD28, have shown promising in efficient TCR activation and expansion of T cells (Mi et al., 2018). Overall, these findings demonstrate the critical role of aAPC geometry in governing the contact surface area between aAPCs and T cells to enhance cancer immunotherapy. Therefore, the development of aAPCs with optimized geometry and surface area of contact holds great promise for improving T cell activation and expansion.

Additionally, the spatial arrangement of TCR complexes is critical for TCR triggering, as has been evidenced (Mossman et al., 2005; Cai et al., 2018). The spatial arrangement of TCRs is the confirmation of their response to stimulus signals (Dustin and Groves, 2012; Goyette et al., 2019). To investigate the connection between transverse ligand spacing and T cell activation, researchers have applied ligand nanopatterning techniques. For instance, Spatz’s group found that the antibodies to CD3 had a transverse spacing of less than 60 nm for optimal T cell activation, but the transverse spacing of pMHC was up to 150 nm, indicating that a spatial requirement is being for TCR priming (Deeg et al., 2013; Matic et al., 2013).

**FIGURE 6:**
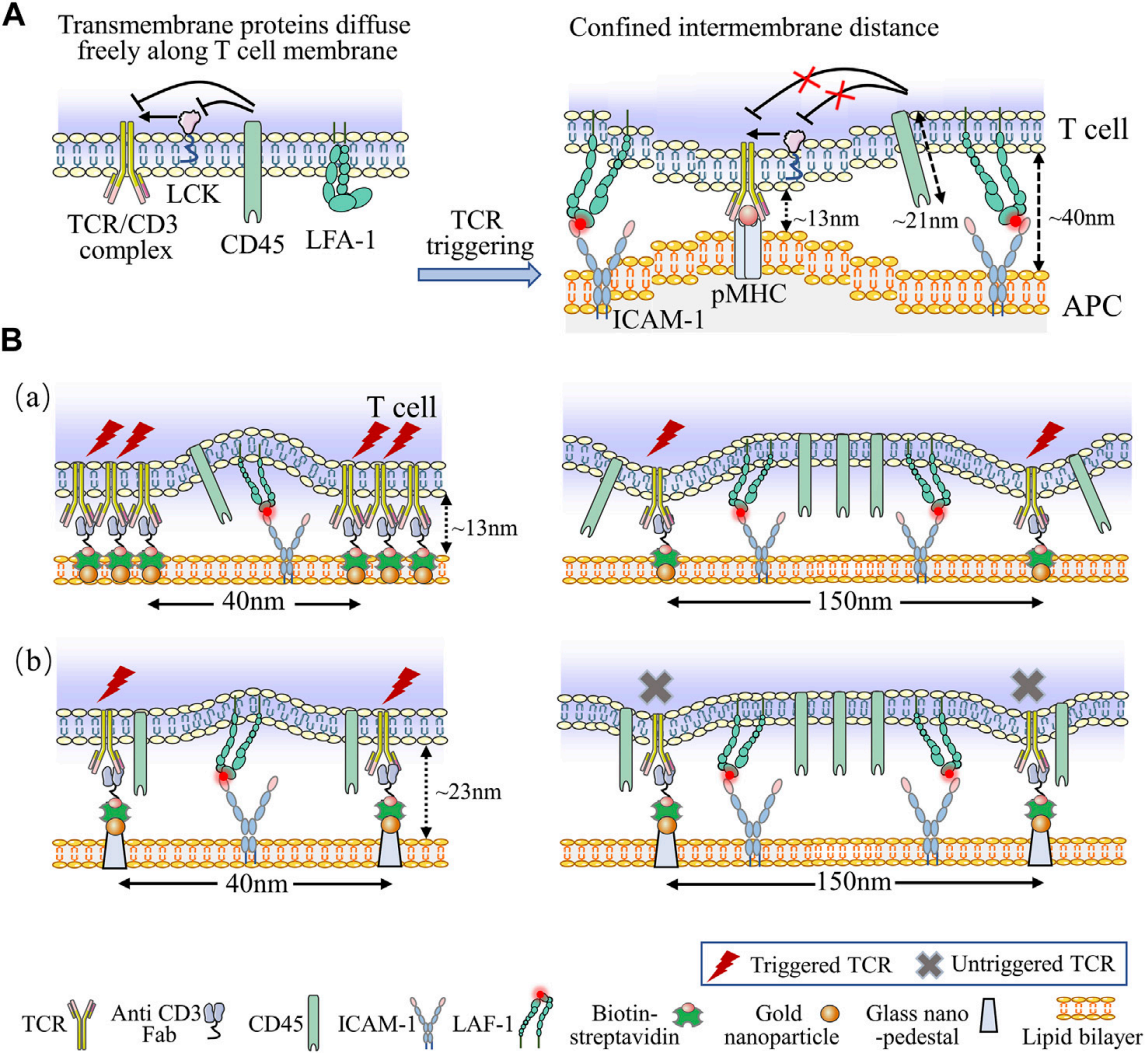
Kinetic models associated with T cell activation. **(A)** The KS model describes the kinetics of T cells activation. At the resting state, transmembrane proteins of T cell can freely distribute across the plasma membrane. The phosphorylation and dephosphorylation of downstream signals of TCR are equilibrated by LCK and CD45, respectively. When TCRs/pMHCs engagement, the intermembrane distance is limited to 13 nm, which physically excludes the proximity of bulky CD45, leading to elevated phosphorylation level and T cell activation. Additionally, bent and inactive LFA-1 is expressed on naïve T cell, upon TCR ligating to ICAM-1, LFA-1 alters its conformation to the low-affinity form, this engagement facilitates the bent form of LFA-1 opening to high-affinity form, which generates the membrane tension with a spacing of 40 nm around TCR/pMHC complexes, providing defined molecular forces leading to membrane bending. **(B)** The schematic diagrams of TBM model. Nanofabricated surfaces are immobilized using gold nanoparticles (NPs) at the SLB (a) or using gold NPs on SiO_2_ nanoarrays exceeded 10 nm above the SLB (b). The anti-CD3 Fab is attached to the NPs and ICAM-l as adhesion molecules exist on SLBs directly. In order to accommodate the large ICAM-1/LFA-1 complexes, the conjugated TCR clusters produces a large membrane curvature (a), while the anti-CD3 Fab with a certain elevation and spacing generates a small membrane curvature (b). Nevertheless, reducing the spacing of nanoarrays to 40 nm recovers TCR triggering signaling (b, left). The force of ICAM-1/LFA-1 interactions is too weak to generate adequate membrane curvature for triggering TCR signaling when the spacing of nanoarrays are 150 nm (b, left).

The performance of artificial substrates loading with nanopatterned antibodies and optimized transverse spacing have surpassed traditional dynabeads, indicating that ligand patterning may be a useful tool for engineering more efficient aAPCs for *in vitro* expansion of T cell (Guasch et al., 2018). This is supported by the Kinetic-Segregation (KS) model, which mentions the limitation of TCR ligands at the nanoscale is essential to the triggering of T cell activation (Choudhuri et al., 2005). Additionally, in the KS model, the phosphorylation and dephosphorylation of zet-chain-associated protein kinase of 70 (Zap-70), an key kinase of TCR signal downstream, are equilibrated by lymphocyte-specific protein tyrosine kinase (LCK) and CD45, respectively, at the resting state (Davis and van der Merwe, 2006). Thus, upon TCRs/pMHCs engagement, the intermembrane distance is limited to approximately 13 nm, which physically excludes the proximity of bulky CD45, leading to elevated phosphorylation of Zap-70 and T cell triggering (Carbone et al., 2017). Notably, the transverse spacing under 50 nm (the actual spacing was 40 nm) rescued the inhibition of T cell triggering, even with the nano-pedestal pattern, indicating that the physical separation of CD45 from the TCR-ligand complexes needs the transverse TCR clustering with less than 50 nm spacing to trigger T cell responses (Figure 6A) (Cai et al., 2018). This model demonstrates a vital parameter for engineering aAPCs to enhance T cell behavior. The TCR bending mechanosignal (TBM) model propose that localized T cell membrane bending at the nanoscale makes constant binding between pMHC and TCR to formulate small complexes which distribute among large surface molecules (CD45) and adhesion molecules (LFA-1) on T cells (Figure 6B) (Al-Aghbar et al., 2022). The TBM model suggests that immunomodulators capable of altering the softness of T cell membranes, such as cholesterol-derived and lipid-soluble compounds which alter lipid composition under aging and pathological conditions, and could lead to new designs for adoptive T cell therapy.

The approaches currently used for activating and expanding T cells *ex vivo*, which take into consideration the T cell behaviors to the surface mechanical properties of materials, such as stiffness, topography and ligand presentation, as summarized in Table 2.

**TABLE 2:** Engineered materials platforms for T cell activation and expansion.

Platform	Features	Application	References
Stiffness	Protein-loading microbeads made from a soft elastomer - polydimethylsiloxane (PDMS)	Enhanced T cell expansion	Lambert et al. (2017)
	0.5–100 kPa poly-acrylamide (PA) hydrogels	T cell exhibits a biphasic response to substrate stiffness via TCR	Yuan et al. (2021)
	4–40 kPa RGD-modified alginate (Alg) hydrogel	Stiffer hydrogel enhances T cell activation in contrast to the softer or 2D materials	Majedi et al. (2020)
	An artificial T cell stimulating matrix engineered with hyaluronic acid (HA) hydrogel	Stimulus signals binding to the engineered matrix can successfully activate CD8^+^ T cell *ex vivo*, whereas soluble signals are much less effective	Hickey et al. (2019)
Topography	Nanoparticles with different shapes	Particle geometry can induce a variety of interactions with CD8^+^ T cells and enhance T cell activation *in vitro* and *ex vitro*	Sunshine et al. (2014)
	Bundled carbon nanotubes attached by antigens are combined with polymer nanoparticles containing magnetite and the T cell growth factor IL-2	Promoted generation of abundant cytotoxic T cells through providing a high density of surface stimulation	Fadel et al. (2014)
	ZnO nanowires with various lengths functionalized by antibodies to CD3	Promoted T cell activation with increasing nanowire length	Bhingardive et al. (2021)
	PDMS micropore arrays with different depth	T cells sense the depth of micropore by the extension of actin-rich invadosome-like protrusions	Huse, 2019; Jin et al. (2019)
Ligand presentation	Artificial antigen presentation surfaces constructed from patterned micro-dot arrays surrounded by a fluid supported lipid bilayer	Useful tools for mimicking pMHC nanoclusters	Goyette et al. (2019)
	Engineering of ligands on planar surfaces with different ligand spacing or patterning	Nanoscale engineering of ligand presentation exerts geometric constraints on the formation of immune synapses	Tabdanov et al. (2015), Cai et al. (2018), Zhang J et al. (2021)

### 3.3 Application of biomechanical forces technology in T cell-mediated cancer immunotherapy

#### 3.3.1 Nanopatterned substrates

Novel precisely nanopatterned substrates have been fabricated for probing TCR/CAR nanospacing and clustering for quantitatively analyzing the effects of ligand nanospacing on the activation of TCR/CAR immunoreceptor. In addition, 2.5D substrates with well-controlled both lateral and vertical nanoscale properties allow the fine tuning of the surface topography that better mimics the complexity of IS interface and microenvironments encountered by CAR-T cell (De la Zerda et al., 2018). Furthermore, these 2D and 2.5D nano-topographic substrates can be integrally incorporated within 96-well plates as a high-throughput mechanobiology screening platform (Hu et al., 2016), it could potentially reveal the complex effects of substrate mechanical properties, surface topography, and biochemical factors on both T cell activation and functional cytokine production, which may have direct clinical applicability in adoptive immunotherapy.

#### 3.3.2 3D scaffold and organoid platforms

Currently, the 3D scaffold platforms are used to analyze the biophysics of IS mechanotransduction, facilitating the understanding of T cell interactions with the complex immune microenvironment. The emerging 3D engineered platforms are contributing to the design of synthetic lymphoid organoids by generating a favorable microenvironment resembling the natural immune tissue niche. A lymph node organoid scaffold made from the mesoporous silica microrods functionalized with pMHC, anti-CD3, and anti-CD28 antibodies effectively enhanced the activation and persistence of T cells embedded in the endonode organoid scaffold (Cheung et al., 2018). Based on the impacts of ECM on T cell, a novel lysyl oxidase (LOX) target strategy was used to modify the mechanotransduction and clinical efficiency of T cell by altering the mechanical properties of the microenvironments which can be widely used on various platforms *in vivo* and *ex vivo* (Nicolas-Boluda et al., 2021). The development of these 3D scaffolds and organoid systems and protocols gives valuable insights into the mechanoimmunology of CAR-T cells, which is truly physiologically relevant.

#### 3.3.3 Microfluidic measurement platforms

The microfluidic platform is an excellent platform for studying mechanoimmunology with an immune organ on chip, which can potentially provide better physiological relevant microenvironment *ex vivo*. For example, the microfluidic lymph node on a chip offers a biomimetic platform for studying how the mechanical signals within the lymph node affect the interaction of APC-T cells (Gopalakrishnan et al., 2015). Similarly, the measurements of the affinity and mechanotransduction of CAR-antigen complexes and the real-time monitoring of interactions of CAR-T cell and tumor in a biomimetic environment *ex vivo* with novel microfluidic-based microphysiological systems would facilitate the rapid screening of the CAR-T cell subsets with the appropriate mechanical properties and highest cytotoxicity, which can dramatically boost the therapeutic efficacy of the CAR-T cell-based immunotherapy.

#### 3.3.4 Single-molecule fluorescence resonance energy transfer (FRET) mechanosensors

The FRET-based mechanosensors have been highlighted for their capacity to probe *in vivo* spontaneous dynamics of subcellular forces and mechanosensitive proteins as well as the compatibility for applications in 3D microenvironment. In addition to serving as a mechanosensor, FRET can also act as mechano-switch tools with pulsed ultrasound or light stimulations that noninvasively control genes for activation and activate inducible CAR-T cells for providing a precise cancer immunotherapy (Pan et al., 2018; Huang et al., 2020). The highly spatiotemporally resolved FRET probes offer a promising possibility in visualizing and modulating the activation of CAR-T cell, which is highly compatible with the rapid change of mechanotransduction events during the activation of CAR-T cell and the cytolysis of tumor.

In conclusion, these fundamental studies discussed above suggest that taking comprehensive consideration of stiffness, high aspect ratio and ligand spatiality into aAPCs makes it possible to maximize the availability between ligands and TCR, improve the quality as well as quantity of desired T cell phenotypes, and optimize the outcome of T cell responses for the ACT. There have been increasing researches on the mechanoimmunology of the CARs, which is conducted from the molecular, cellular, and clinical levels. Novel technologies to explore the mechanobiological mechanisms for CAR-T cell activation and tumor cytolysis are critical in achieving the efficacy of immunotherapy.

## 4 Outlook

Mechanical immunoengineering of T cells is an emerging research direction in immunotherapy, where biomechanical cues are employed to manipulate T cells. However, the lack of comprehensive perception on the effects of T cells mechanosensing under pathophysiological states presents a major challenge for the development of mechanical immunoengineering approaches. While ex vivo researches have provided crucial opinions into T cell responses to mechanical signals, they usually cannot entirely replicate actual cell-to-cell behaviors and three-dimensional viscoelasticity in vivo. Furthermore, our understanding of T cell mechanobiology, including mechanotransduction pathways and mechanical forces-biochemical signal interactions to modulate T cell function, remains limited. Therefore, more efforts are needed to broaden our understanding of T cell mechanobiology.

Despite the challenges in understanding T cell mechanobiology, knowledge gained from previous fundamental research efforts have inspired us to integrate biomechanical principles into the immunotherapies design of T cells. One promising approach is the design of dynamic platforms that can control the mechanical checkpoint (YAP, NFAT-1, MRTF) through varying mechanical signals in the microenvironment based on mechanosensitive receptors to boost T cell with desired phenotypes. As known to us, mechanical forces could regulate the activation of T cells, which makes it possible for stiffness of cancer cells to affect T cell cytotoxic, and to be a therapeutic target. Another potential application is the alteration of cancer cell’s mechanical properties to enhance the response of cytotoxic T cells, which could be used in combination with standard chemotherapies and immunotherapies, such as antibodies to PD-1/PD-L1/CTLA-4 for checkpoint blockading. The understanding of T cell mechanosensing and actin function has direct clinical implications. As we better understanding how actin regulators alter mechanical response of T cell, it has been more promising to target actin kinetics to affect T cell activation and differentiation. Hence, the T cell mechanical behaviors could serve as a mark for drug screening by targeting the initial activity of T cell. In addition, the ability to mechano-initiate CAR-T cells to obtain self-penetrating ECM from dense solid tumors would be a promising approach for T cell immunoengineering to enhance the efficacy of CAR-T therapy.
